# Clustering and machine learning techniques identify air pollution regimes in Greater Cairo

**DOI:** 10.1038/s41598-026-49777-5

**Published:** 2026-05-02

**Authors:** Doaa M. Elmourssi, A. M. El-Assy, Hanan M. Amer

**Affiliations:** Electronics and Communications Engineering Department, Faculty of Engineering , Mansoura University, Mansoura, 35516 Egypt

**Keywords:** Air pollution, Machine learning, Urban environments, K-means clustering, Random forest, Decision trees, Greater Cairo, Climate sciences, Environmental sciences, Environmental social sciences, Mathematics and computing

## Abstract

Air pollution is a major environmental and public health challenge in Greater Cairo due to rapid urbanization, intense traffic activity, and recurrent regional dust intrusions. This study presents a data driven analytical framework for identifying and interpreting recurring air pollution regimes in the city. The framework combines unsupervised K-means clustering with supervised Decision Tree (DT) and Random Forest (RF) models using atmospheric reanalysis data derived from the Copernicus Atmosphere Monitoring Service (CAMS) for the period 2023–2024. The optimized K-means model identified four distinct pollution regimes. Low pollution conditions accounted for approximately 75.1% of the analyzed period, whereas higher pollution regimes were mainly associated with traffic related emissions and episodic dust events. To assess the separability of the identified regimes, DT and RF classifiers were trained to predict cluster membership. The optimized Decision Tree achieved an accuracy of 93.10%, while the Random Forest model showed better classification performance, reaching a maximum accuracy of 97.49%, with a practical optimum of 97.43% obtained using 300 trees. Feature importance analysis showed that NO₂ was the dominant variable for distinguishing traffic related pollution regimes, whereas PM₁₀ played a key role in identifying dust related events. Overall, the findings indicate that integrating clustering with tree based classification provides an interpretable and effective approach for characterizing urban air pollution patterns. The resulting framework may support regime based air quality interpretation and targeted management strategies in Greater Cairo.

## Introduction

Air pollution poses a severe and persistent environmental and public health challenge in Greater Cairo, where concentrations of fine particulate matter (PM₂.₅) regularly exceed World Health Organization guidelines by 250–300%^1^. The combined effects of rapid population growth, intensified industrial activity, and expanding urban infrastructure have created intricate pollution patterns that are difficult to decipher using traditional monitoring techniques alone^2^. These complexities are exacerbated by seasonal meteorological conditions and frequent regional dust events, which introduce substantial variability into the city’s air quality dynamics.

Although machine learning methods are increasingly used in air quality research, existing studies often emphasize predictive modeling or source apportionment, with less emphasis on regime based pattern classification and interpretation. For example, recent work employing nonlinear and topological techniques has emphasized pollutant contribution analysis without extending the framework toward operational regime identification or early warning applications^3^. Conversely, Chen and Li (2024) focused on identifying pollution related associations but did not integrate predictive modeling^4^. This dichotomy between descriptive and predictive analysis limits a holistic understanding of how pollution drivers interact in cities such as Cairo, where traffic emissions, industrial sources, and desert dust dynamics frequently coincide^5^. In this context, the present study applies an interpretable analytical framework that combines unsupervised clustering and supervised tree based machine learning techniques to identify and characterize recurring air pollution regimes in Greater Cairo. Using atmospheric data derived from the Copernicus Atmosphere Monitoring Service (CAMS) for 2023–2024^6^, the analysis is designed to support both pattern discovery and regime classification within a consistent multivariable framework. This combined approach is particularly relevant in complex urban environments where emissions, industrial activity, meteorological variability traffic, and desert dust may interact over multiple temporal scales^5,8^.

The contribution of the present study lies in combining established clustering and classification techniques within an interpretable workflow for regime based air pollution analysis^7^. In particular, the study provides a data driven characterization of recurring pollution patterns in Greater Cairo using CAMS derived variables and evaluates the extent to which these regimes can be distinguished using supervised classification models. The resulting analysis may support air quality interpretation and provide a useful basis for future monitoring and early warning efforts in complex urban settings^9,10^.

The remainder of this paper is organized as follows. Section 2 reviews related work on machine learning applications in air quality studies. Section 3 describes the study area, dataset, and analytical methodology. Section 4 presents and discusses the experimental results. Finally, Sect. 5 concludes the paper and outlines directions for future research.

## Literature review

Machine learning (ML) has established itself as a central component of modern environmental monitoring systems, particularly in urban air quality assessment. Earlier research relied heavily on traditional statistical tools, such as linear regression and correlation analysis; however, these methods often fail to adequately capture the nonlinear interactions and multivariate dependencies inherent in atmospheric processes^11^. The advent of high resolution environmental datasets has propelled ML methods to the forefront as more capable alternatives for modeling complex pollution dynamics.

Among unsupervised techniques, K-means clustering has been widely used to uncover hidden structures in air quality datasets. For instance, Wang and Chen demonstrated that clustering can effectively segregate pollution episodes driven by meteorological and seasonal variability, thereby providing valuable descriptive insight into urban air quality regimes^12^. Despite their utility for pattern discovery, clustering methods alone lack predictive capability and offer limited interpretability in the absence of complementary modeling techniques.

In the supervised learning domain, algorithms such as Decision Trees (DT) and Random Forests (RF) have demonstrated strong performance in air quality prediction tasks. Liu et al. highlighted that tree based models tend to surpass classical statistical approaches owing to their ability to capture nonlinear relationships and handle heterogeneous environmental data^13^. Random Forests, in particular, enhance robustness through ensemble learning and have proven effective for high dimensional atmospheric datasets. A recurring limitation, however, is that most existing studies apply these models in isolation rather than as part of an integrated analytical framework^14^.

In recent years, several studies have implemented hybrid or ensemble machine learning frameworks for high resolution air quality inference in large urban environments. For example, Song et al. (2021) proposed the Multi AP learning network to estimate multi pollutant concentrations at 1 km × 1 km hourly resolution using convolutional architectures and multi view feature representations^15^. Similarly, Deep MAPS employed mobile and fixed sensor integration with deep learning models to achieve fine grained PM₂.₅ mapping in Beijing^16^. The MCST Tree framework introduced a multi cascade gradient boosting structure to enhance spatial temporal PM₂.₅ inference performance^17^. NetGBM further extended tree based ensemble modeling to large scale ozone prediction with integrated meteorological and land use features^18^. More recently, the ST Exposure framework demonstrated pixel level inhalation exposure modeling using multi source data fusion and gradient boosting methods^19^.

While these studies achieve remarkable spatial temporal inference accuracy, they primarily focus on concentration estimation or exposure reconstruction rather than pollution regime discovery and classification. Moreover, a considerable portion of recent high resolution hybrid ML frameworks has been developed and evaluated in East Asian megacities, particularly in China, where dense monitoring and mobile sensing infrastructures are available. While such approaches are increasingly explored globally, regime based clustering classification frameworks in arid Middle Eastern megacities using reanalysis driven datasets such as CAMS remain comparatively underexplored.

Collectively, the existing body of work underscores substantial progress in applying ML to air quality management. Notwithstanding these advances, a salient gap persists: the scarcity of hybrid frameworks that cohesively integrate unsupervised pattern discovery and supervised classification within a systematically optimized analytical framework.

Directly targeting this gap, the present study implements an optimized hybrid framework that combines elbow optimized K-means clustering with hyperparameter tuned Decision Tree and Random Forest models within a unified analytical workflow designed for CAMS atmospheric datasets^20^. This integrated approach is designed to deliver a more comprehensive and interpretable understanding of Cairo’s air pollution dynamics by linking pattern discovery with supervised regime classification. To clearly position the present framework within the existing body of literature, Table 1 provides a comparative summary of recent hybrid machine learning approaches for urban air quality modeling.

**Table 1:** Comparative summary of recent hybrid machine learning frameworks for urban air quality modeling.

Study	Study area	Data sources	ML framework	Target output	Resolution	Key predictors	Reported performance
Song et al. (2021)^15^	Chengdu, China	Fixed stations & urban features	Multi-AP (CNN-based multi-output network)	Multi-pollutant concentration	1 km × 1 km, hourly	Meteorology, land use	High accuracy (multi-output inference)
Deep-MAPS (2021)^16^	Beijing, China	Fixed & mobile sensors	Deep learning framework	PM₂.₅ concentration	1 km × 1 km, hourly	Urban big data, traffic	SMAPE < 15%
MCST-Tree (2022)^17^	Chengdu, China	Fixed & mobile sensors	Multi-cascade GBDT ensemble	PM₂.₅ concentration	1 km × 1 km, hourly	Land use, meteorology	R² ≈ 0.94
NetGBM (2024)^18^	China (national scale)	Fixed stations + meteorology	Gradient boosting ensemble	O₃ concentration	Daily–monthly grids	Temperature, land use	R² = 0.77–0.83
ST-exposure (2024)^19^	Beijing, China	Fixed + mobile + population mobility	GBDT-based exposure model	PM₂.₅ inhalation volume	1 km × 1 km, hourly	Population mobility, meteorology	SMAPE < 15%
Present study	Greater Cairo, Egypt	CAMS reanalysis	K-means + DT + RF (hybrid clustering–classification)	Pollution regime discovery & classification	Daily scale	PM₂.₅, PM₁₀, NO₂ + meteorology	DT: 93.1% RF: 95.38%

## Methodology

**Fig. 1:**
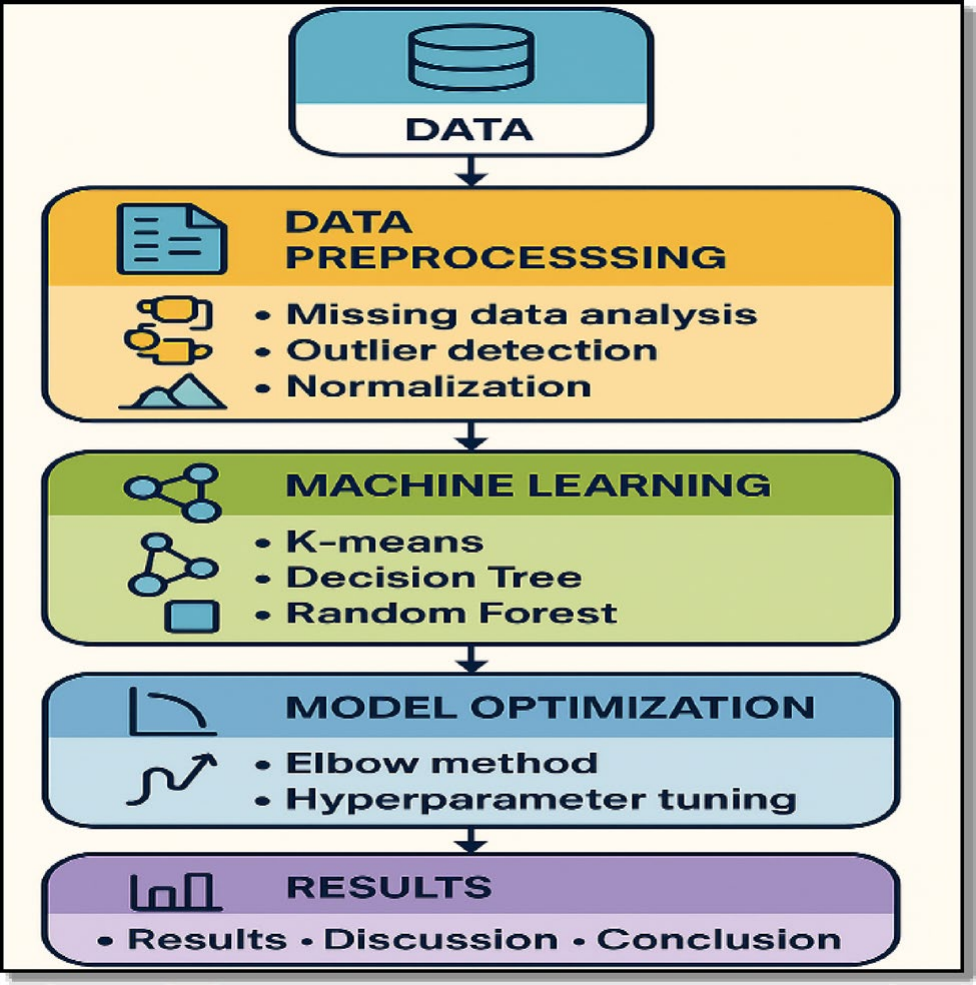
Workflow of the proposed machine learning framework for air quality analysis.

Before describing each methodological component in detail, an overview of the complete workflow is presented in Fig. 1.

### Study area and data source

The Greater Cairo Metropolitan Area is one of the most polluted megacities in the Middle East, with a population exceeding 20 million inhabitants. Air quality in this region is strongly influenced by dense traffic, concentrated industrial activity, and recurrent seasonal dust storms transported from surrounding desert regions^21,22^.

Data used in this study were obtained from the Copernicus Atmosphere Monitoring Service (CAMS)^23^, which provides global reanalysis fields generated through advanced data assimilation systems that combine satellite observations, in situ measurements, and chemical transport modelling^24^. The CAMS products offer high spatial and temporal consistency, making them particularly suitable for data driven air quality applications.

The dataset covers a continuous two year period from January 2023 to December 2024 and includes both pollutant and meteorological variables: PM₂.₅, PM₁₀, NO₂, SO₂, O₃, CO, near surface air temperature, dew point, wind speed, and surface pressure. CAMS data were extracted at a 3 hourly temporal resolution over the Greater Cairo region and subsequently quality controlled prior to analysis. Following preprocessing, a total of 5,848 complete records were retained, providing a robust basis for subsequent data mining and predictive modelling tasks^25,26^. It is important to acknowledge the limitations associated with using CAMS reanalysis data in complex arid urban environments such as Greater Cairo. Previous global validation studies have shown that while CAMS reanalysis products provide consistent large scale atmospheric variability, regional biases may persist when compared against ground based observations, particularly over dust prone and arid regions. These uncertainties are primarily related to aerosol speciation and coarse mode dust representation and may affect absolute concentration estimates (Gueymard and Yang, 2020)^27^. In this study, CAMS data are not treated as point wise ground observations but rather as a spatially and temporally consistent representation of regional atmospheric variability. Accordingly, the proposed framework focuses on identifying recurring air pollution regimes and relative multi variable patterns rather than relying on absolute concentration thresholds. This regime oriented perspective is inherently more robust to systematic reanalysis biases and remains suitable for supporting short term situational awareness and decision making in data sparse urban environments.

### Data preprocessing

To ensure the reliability of the dataset and enhance subsequent model performance, a structured data preprocessing pipeline was implemented^24^. The procedure consisted of three main stages: (i) missing data assessment, (ii) outlier detection and treatment, and (iii) normalization.

#### Missing data analysis

The CAMS reanalysis dataset showed no missing values for any of the selected pollutant or meteorological variables^24^. This completeness reflects the strength of the CAMS assimilation system, which combines satellite and ground based observations to maintain temporal continuity. As a result, the dataset was used directly without applying any imputation procedures, preserving the original information content and avoiding potential artefacts introduced by synthetic values.

#### Outlier detection and treatment

Outliers were identified using the interquartile range (IQR) method supported by contextual inspection of time series and known pollution episodes as shown in Table 2. The analysis indicated that pollutant variables contained between 4.2% and 8.2% outliers, corresponding mainly to genuine high pollution events, while meteorological parameters exhibited less than 1% extreme values.

**Table 2:** Summary of outlier detection results and treatment actions.

Variable	Outlier %	Action
PM₂.₅	4.2%	Retained
PM₁₀	6.8%	Retained
CO	8.2%	Retained
Wind speed	0.7%	Winsorized
Pressure	0.3%	Winsorized

For pollutant variables, outliers were retained in order to preserve the representation of real extreme events and avoid underestimating pollution severity^25^. For meteorological variables, Winsorizing was applied, replacing extreme values with the nearest acceptable bounds. This reduced the influence of potential measurement errors and improved data stability prior to normalization.

#### Data normalization

All variables were standardized using Z score normalization to ensure a common scale before model training. This method transformed each feature to have zero mean and unit standard deviation according to.

1 $$X\_normalized{\text{ }} = {{\left( {X{\text{ }} - {\text{ }}\mu } \right)} \mathord{\left/ {\vphantom {{\left( {X{\text{ }} - {\text{ }}\mu } \right)} \sigma }} \right. \kern-\nulldelimiterspace} \sigma }$$

Where X is the original value, µ is the mean of the feature, and σ is its standard deviation.

This transformation prevents variables with larger numerical ranges from dominating the analysis and improves the behavior of algorithms that rely on distance calculations, such as K-means clustering, while also contributing to numerical stability for tree based ensemble models during optimization.

### K-means clustering analysis

This subsection outlines the methodology used to apply the K-means algorithm and the procedures followed to identify coherent pollution regimes within the dataset.

#### Standard K-means implementation

K-means clustering, an unsupervised machine learning algorithm, was applied to identify recurrent air pollution regimes within Cairo’s air quality dataset. The analysis incorporated standardized pollutant concentrations alongside relevant meteorological variables to capture both emission related and dispersion related influences on air quality. Prior to clustering, all features were standardized using Z score normalization to ensure equal contribution to the Euclidean distance metric. The K-means algorithm partitions observations into k clusters by iteratively minimizing the within cluster sum of squared distances, thereby producing compact and well separated groups in the feature space^28^. This iterative process continues until cluster centroids converge or a predefined stopping criterion is satisfied. MATLAB’s built in kmeans function was employed using multiple random initializations (replicates) to enhance solution stability and reduce sensitivity to centroid initialization. This implementation follows established practices in environmental data mining applications and ensures reproducible and robust clustering outcomes^29,30^.

#### Model optimization using the Elbow method

**Fig. 2:**
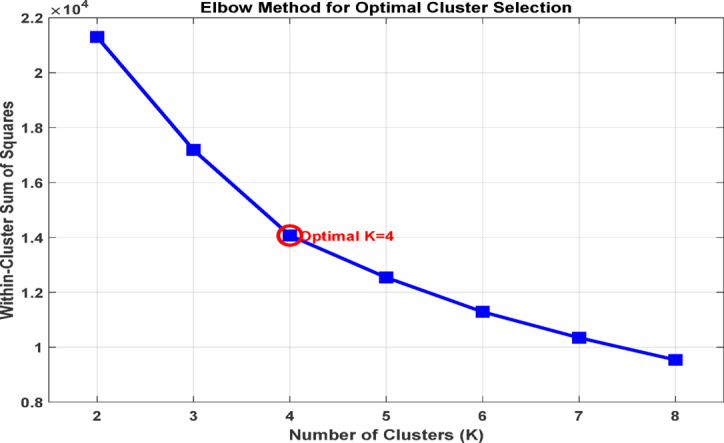
Elbow plot showing the optimal number of clusters (k = 4).

To determine an appropriate number of clusters for the K-means analysis, the Elbow Method was applied by evaluating the within cluster sum of squares (WCSS) across a range of candidate values of k. This approach assesses the tradeoff between cluster compactness and model complexity by examining how the total within cluster variance decreases as the number of clusters increases^31^.The analysis revealed a clear change in slope at k = 4, indicating that additional clusters beyond this point result in only marginal reductions in WCSS. This inflection point suggests that a four cluster configuration provides an effective balance between capturing meaningful structure in the data and avoiding unnecessary model complexity. Selecting four clusters reduces the risk of arbitrary partitioning and supports the identification of physically interpretable air pollution regimes rather than purely algorithmic groupings. The resulting configuration therefore provides a consistent and interpretable foundation for subsequent regime characterization and supervised classification analyses^32,33^. Figure 2 illustrates the Elbow curve, where the observed change in slope at k = 4 supports the selected number of pollution regimes.

#### Cluster validation and robustness assessment

Although the Elbow method provides a useful heuristic for determining the optimal number of clusters, it does not constitute a formal statistical validation criterion. Therefore, additional complementary cluster validation metrics were computed to assess the robustness of the selected four cluster configuration.

Specifically, three widely used internal validation indices were evaluated for k values ranging from 2 to 8:

- The Silhouette score, which measures the degree of separation and cohesion among clusters^34^.
- The Calinski–Harabasz (CH) index, which evaluates the ratio of between cluster dispersion to within cluster dispersion^35^.
- The Gap statistic, which compares observed within cluster dispersion to that expected under a reference null distribution^36^.

**Fig. 3:**
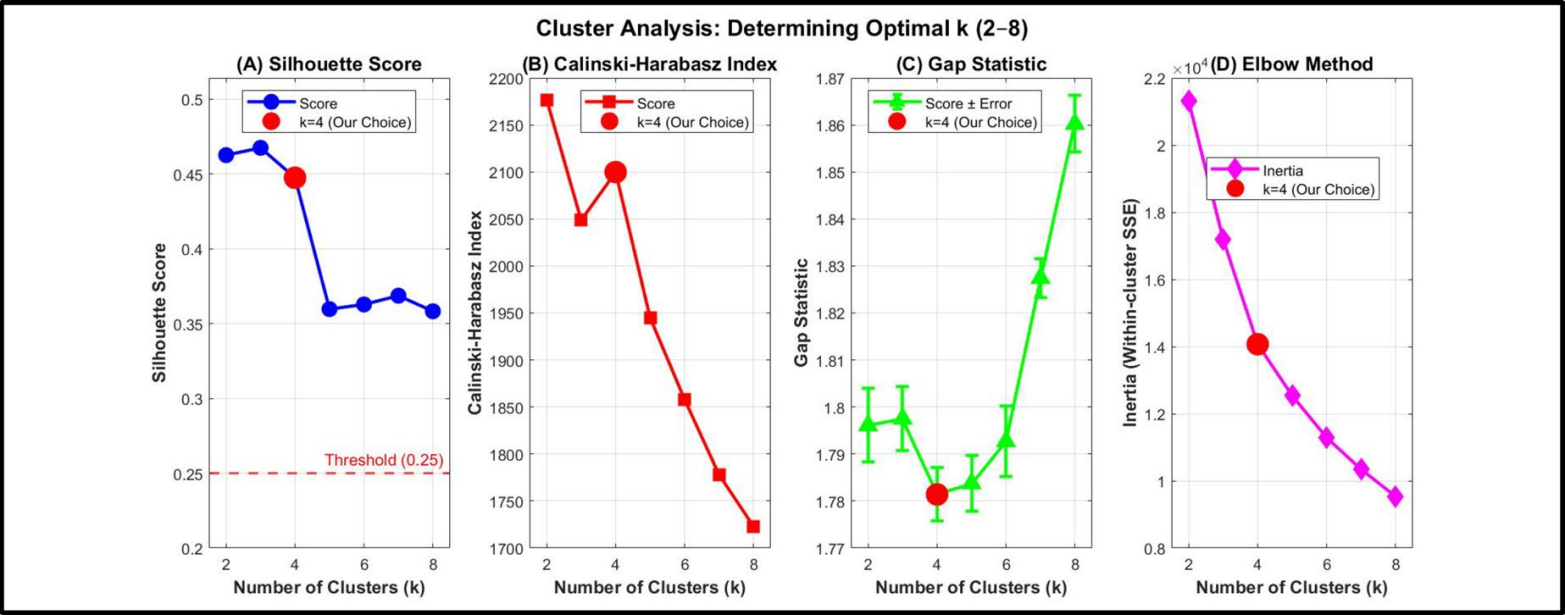
Multi metric cluster validation for k = 2–8 using Silhouette score, Calinski–Harabasz index, Gap statistic, and Elbow method.

All validation curves are presented in Fig. 3. The Silhouette scores remained above 0.25 across all tested k values, indicating meaningful clustering structure. For k = 4, the Silhouette score was 0.388, reflecting acceptable cluster separation. The Calinski–Harabasz index for k = 4 reached 2099.8, indicating strong inter cluster separation and compactness. Additionally, the Gap statistic remained positive across all candidate k values, confirming that the identified structure deviates significantly from random clustering.

Although different validation metrics may peak at slightly different k values a common phenomenon in unsupervised learning the four cluster configuration demonstrates consistent statistical support while preserving clear environmental interpretability for Greater Cairo’s pollution regimes. Accordingly, k = 4 was retained.

To further assess temporal robustness and regime recurrence, a 50% temporal validation experiment was conducted. K-means clustering was first trained using only 2023 data, and the resulting centroids were subsequently applied to independently assign cluster labels to 2024 observations. Agreement between predicted and independently derived 2024 clusters was quantified using the Adjusted Rand Index (ARI)^37^, which measures similarity between two partitions while correcting for chance agreement.

The ARI value reached 0.826, indicating strong structural agreement between 2023 and 2024 cluster configurations (ARI ranges from − 1 to 1, where 1 denotes perfect agreement and 0 corresponds to random labeling). This result confirms that the four identified pollution regimes represent stable and recurring atmospheric patterns rather than year specific artefacts.

### Decision tree classification

This section presents the supervised classification framework based on Decision Trees, which was employed to evaluate the consistency and separability of the pollution regimes identified by the K-means clustering analysis. In addition to assessing regime separability, the Decision Tree model provides an interpretable rule based structure that links environmental variables to the four identified air pollution regimes.

#### Base decision tree model

The Decision Tree (DT) algorithm was implemented as a supervised learning approach to evaluate the internal consistency of the regimes derived from the optimized K-means clustering and to develop an interpretable framework for regime membership classification. The model was trained using the standardized dataset, which included PM₂.₅, PM₁₀, NO₂, SO₂, O₃, CO, near surface air temperature, wind speed, and surface pressure as input features^38^.Cluster assignments obtained from the optimized K-means algorithm were used as categorical target labels, enabling the DT model to learn decision rules that associate specific combinations of atmospheric conditions with each of the four identified pollution regimes.

#### Hyperparameter optimization process

A systematic hyperparameter optimization procedure was conducted to improve the predictive performance and generalization capability of the Decision Tree model^39^. Decision Trees provide a high degree of interpretability by mapping threshold based rules between environmental variables and pollution regimes, which is particularly valuable for environmental assessment and decision support applications^40^.

To control model complexity and reduce the risk of overfitting, a range of tree depths was evaluated. Specifically, fourteen maximum depths were tested (10, 15, 20, 25, 30, 40, 50, 60, 70, 80, 90, 100, 120, and 150) to identify an appropriate balance between model expressiveness and stability. In addition to depth optimization, further hyperparameters were examined, including the minimum leaf size (1, 5, 10, and 20) and alternative splitting criteria. These configurations were assessed to determine the most suitable tree structure for capturing the variability of air pollution regimes in Greater Cairo. Model performance was evaluated using a stratified data partitioning scheme, with 70% of the samples used for training and 30% reserved for testing^41^, ensuring proportional representation of all pollution regimes in both subsets. Classification accuracy was employed as the primary metric for selecting the optimal Decision Tree configuration, providing a clear basis for comparing model performance across different parameter settings. Although the optimized Decision Tree reached a relatively large maximum depth (90), additional structural diagnostics indicate that the model does not suffer from significant overfitting. The final tree consists of 81 terminal nodes with an average leaf size of 50.54 samples. Furthermore, the training and testing accuracies were 97.36% and 93.10%, respectively, corresponding to a limited train–test accuracy gap of 4.26% points. These indicators confirm that the selected tree depth captures complex relationships in the data while maintaining good generalization performance.

### Random forest classification

This section presents the Random Forest classification framework used to evaluate and predict air pollution regime membership, focusing on the ensemble structure, training configuration, and optimization strategy.

#### Ensemble model framework and configuration

The Random Forest (RF) algorithm was implemented as a robust ensemble learning technique to enhance classification accuracy and model generalization for regime membership discrimination. The model utilized the same standardized input variables as the Decision Tree model, including PM₂.**₅**, PM₁₀, NO₂, SO₂, O₃, CO, near surface air temperature, wind speed, and surface pressure, with K-means cluster assignments serving as categorical target labels^42^.The ensemble was constructed using bootstrap aggregation and random feature sampling to generate multiple weakly correlated decision trees. Model predictions were obtained through majority voting across the ensemble. An initial configuration of 100 trees was adopted, with Out of Bag (OOB) error estimation enabled to provide an internal and unbiased assessment of model performance.

#### Advanced hyperparameter optimization and model validation

A comprehensive hyperparameter optimization procedure was applied to determine the optimal RF configuration for classifying pollution regimes in Greater Cairo. Ensemble sizes ranging from 50 to 500 trees were evaluated, alongside variations in the minimum leaf size (1–20) and the number of predictor variables randomly sampled at each split (3–9 features)^43^. Model performance was assessed using a stratified 70/30 train test split consistent with the Decision Tree evaluation, ensuring balanced representation of all pollution regimes in both subsets. Classification accuracy was employed as the primary selection metric. In addition, permutation based variable importance was computed to quantify the relative contributions of pollutant and meteorological variables to the regime classification process.

## Results and discussion

This section presents the key findings of the optimized machine learning framework, including the identification of air pollution regimes, the performance of the supervised classification models, and a comparative interpretation of the dominant predictive factors influencing air quality dynamics in Greater Cairo.

### Optimized air pollution pattern discovery

The Elbow Method confirmed k = 4 as an appropriate number of clusters, revealing four distinct atmospheric pollution regimes across Greater Cairo. This optimized configuration resulted in clear separation between pollution regimes and improved interpretability compared with conventional clustering approaches. Table 3 summarizes the characteristics of the four clusters based on standardized pollutant and meteorological indicators.

**Table 3:** Optimized K-means clustering results for Greater Cairo.

Cluster	Percentage	PM₂.₅ (std)	PM₁₀ (std)	NO₂ (std)	PM₂.₅ Range (µg/m³)	PM₁₀ Range (µg/m³)	NO₂ Range (ppb)	Primary characteristics
1	33.4%	−0.321	−0.124	−0.637	9.2–25.1	18.5–75.3	1.9–12.0	Low pollution regime
2	41.7%	−0.429	−0.406	−0.146	8.7–23.9	14.9–57.1	3.7–15.8	Very low pollution regime
3	18.9%	0.959	0.208	1.595	16.0–45.3	26.0–84.7	11.5–30.8	Traffic dominated pollution
4	6.0%	1.743	2.845	−0.451	21.3–57.7	79.1–201.5	2.1–13.8	Dust storm pollution

**Fig. 4:**
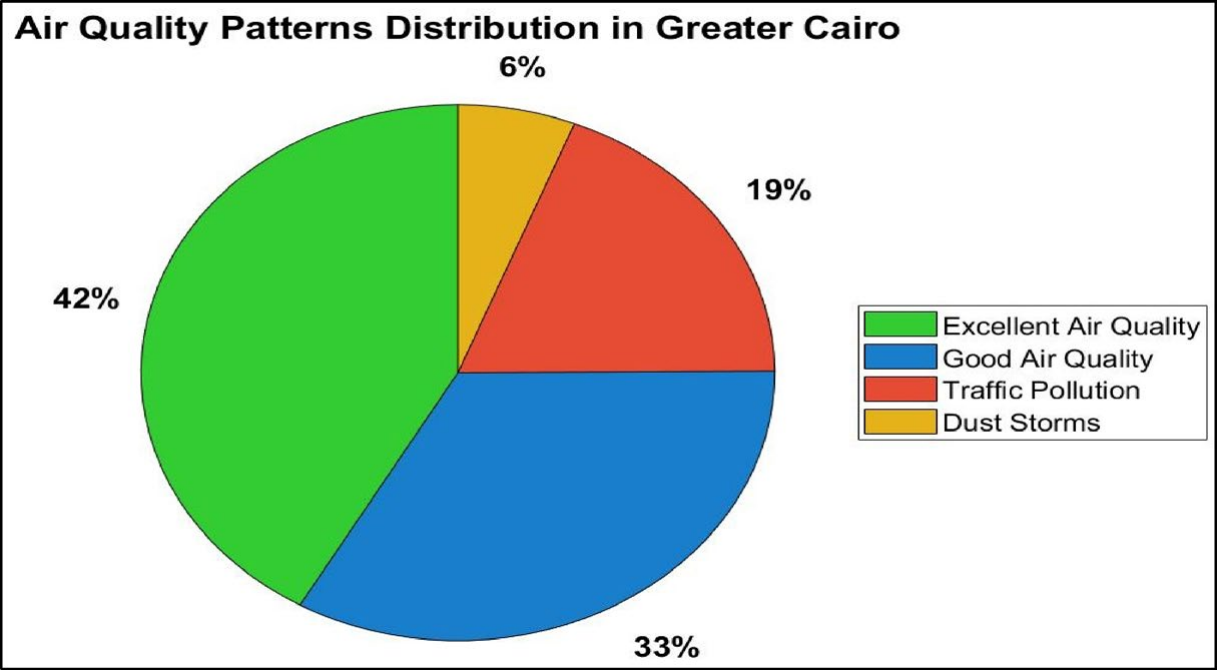
Distribution of the four air quality clusters in Greater Cairo.

The clustering results provide several important insights into Cairo’s air pollution dynamics. First, only 6.0% of the analyzed period corresponds to severe dust storm pollution episodes, characterized by extremely elevated PM₁₀ concentrations reaching 79.1–201.5 µg/m³, exceeding WHO 24-hour guidelines. Second, approximately 75.1% of observations fall within low to very low pollution regimes (Clusters 1 and 2), indicating that favorable atmospheric conditions dominate most of the study period. Third, the clear distinction between dust related pollution (Cluster 4) and traffic dominated pollution (Cluster 3) highlights the presence of distinct emission driven regimes, facilitating improved source attribution and more targeted mitigation strategies. The measured NO₂ concentrations during traffic dominated episodes (11.5–30.8 ppb) exceed WHO annual and 24-hour limits, underscoring the impact of vehicular emissions. Overall, the optimized clustering structure provides a robust foundation for characterizing pollution variability and identifying the dominant atmospheric regimes influencing air quality in Greater Cairo. Figure 4 illustrates the temporal distribution of the four identified pollution regimes.

### Decision tree classification results

This subsection presents the performance of the Decision Tree classifier in distinguishing the air pollution regimes derived from the K-means clustering analysis. The results include model optimization outcomes, classification performance evaluation, and identification of the most influential environmental predictors contributing to regime discrimination.

#### Model optimization and performance

The systematic hyperparameter optimization process led to substantial improvements in Decision Tree classification performance. Classification accuracy increased from 84.38% at a tree depth of 10 to a maximum of 93.10% at an optimal depth of 90, as summarized in Table 4. This represents an absolute improvement of 8.72%, highlighting the importance of appropriate parameter tuning for effective regime membership classification in complex urban air pollution datasets. Performance saturation was observed beyond a depth of 90, with deeper trees (depths ranging from 100 to 150) achieving comparable accuracies between 92.99% and 93.10%. This behavior indicates diminishing returns from further increasing model complexity and suggests that a depth of 90 provides an effective balance between predictive capability and model stability when representing Cairo’s pollution regimes^44^.

**Table 4:** Decision tree classification accuracy across depth variations.

Tree Depth	Accuracy (%)	Improvement
10	84.38	Baseline
20	88.08	+ 3.70%
30	90.02	+ 5.64%
90	93.10	+ 8.72%
150	92.99	+ 8.61%

#### Feature importance and dominant predictors

**Fig. 5:**
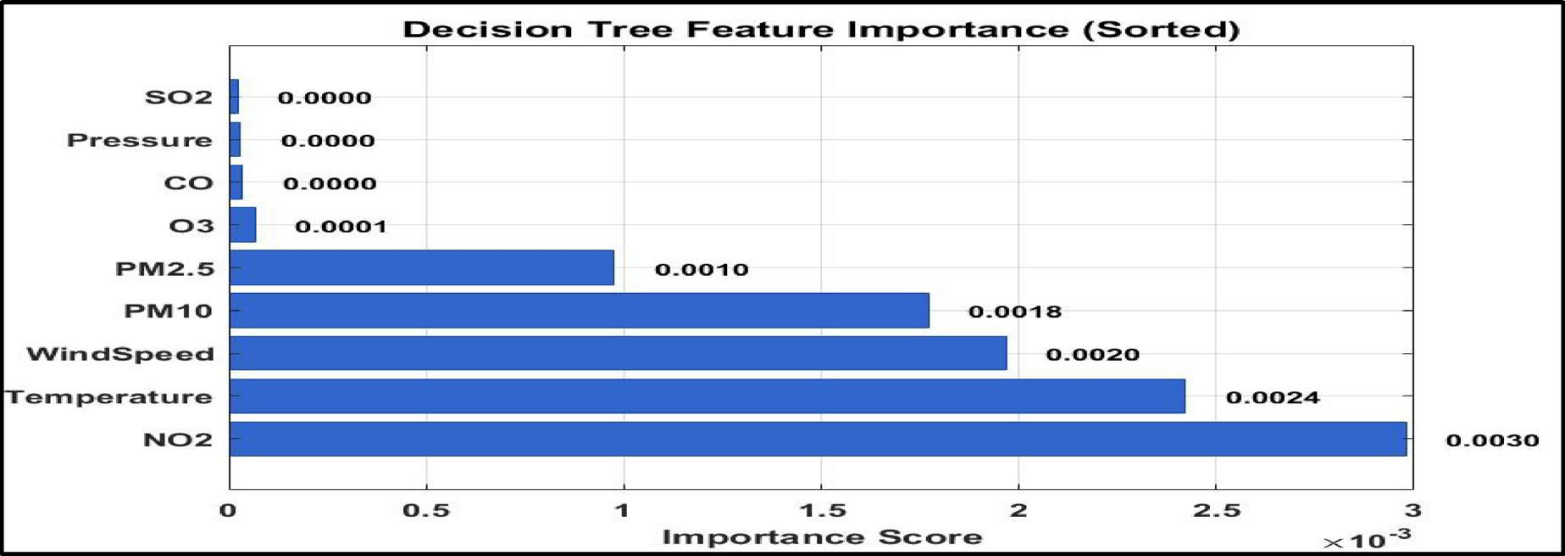
Feature importance in decision tree classification model.

Feature importance analysis, illustrated in Fig. 5, identifies NO₂ as the most influential predictor in distinguishing between the identified pollution regimes, with an importance score of 0.0030. This is followed by near surface air temperature (0.0024) and wind speed (0.0020). These results indicate that traffic related emissions and meteorological conditions jointly play a dominant role in shaping urban air pollution regimes in Greater Cairo^45^. The prominence of NO₂ highlights the substantial contribution of vehicular emissions to traffic dominated pollution regimes, while the influence of temperature and wind speed reflects the critical role of atmospheric stability and dispersion processes in pollutant accumulation and transport^46^. Together, these findings demonstrate that the Decision Tree model captures physically meaningful relationships between environmental variables and the identified pollution regimes.

### Random forest optimization results

This subsection evaluates the performance of the Random Forest classifier for pollution regime classification and examines the impact of ensemble size and hyperparameter tuning on model accuracy. The analysis highlights the performance gains achieved over the baseline Decision Tree model and identifies an optimal Random Forest configuration for distinguishing Cairo’s pollution regimes.

#### Ensemble optimization and performance

Systematic hyperparameter optimization of the Random Forest model resulted in consistently high classification performance across a wide range of ensemble sizes. Classification accuracy increased from 97.15% with 50 trees to a maximum of 97.49% with 450 trees, as summarized in Table 5. A practical ensemble size of 300 trees achieved an accuracy of 97.43%, corresponding to an absolute improvement of 4.33% over the optimized Decision Tree baseline. The performance analysis reveals a clear plateau beyond approximately 300 trees, with ensemble sizes between 50 and 600 trees yielding comparable accuracies ranging from 97.15% to 97.49%. The marginal gain of only 0.06% observed when increasing the ensemble size from 300 to 450 trees indicates diminishing returns from further increasing model complexity. Consequently, an ensemble of 300 trees was selected as the optimal configuration, providing an effective balance between classification accuracy, computational efficiency, and model stability for regime membership classification in Cairo’s urban atmosphere^43,44^.

**Table 5:** Random Forest classification accuracy across ensemble sizes.

Number of trees	Accuracy (%)	Improvement over DT (93.10%)
50	97.15	+ 4.05%
100	97.32	+ 4.22%
150	97.38	+ 4.28%
200	97.21	+ 4.11%
250	97.21	+ 4.11%
300	97.43	+ 4.33%
350	97.15	+ 4.05%
400	97.32	+ 4.22%
450	97.49	+ 4.39%
500	97.32	+ 4.22%
600	97.38	+ 4.28%

#### Enhanced feature importance analysis

**Fig. 6:**
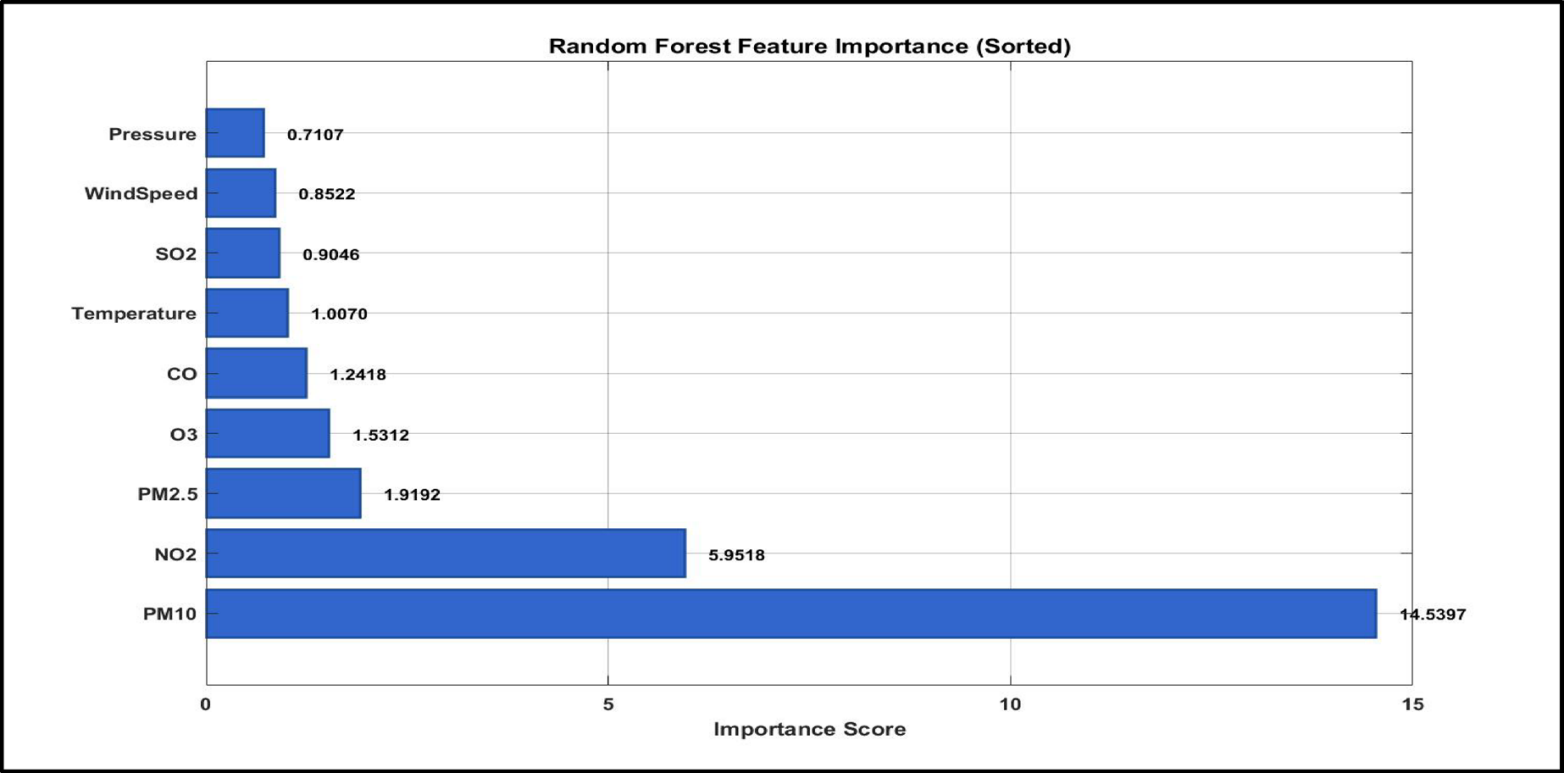
Feature Importance in random forest classification model.

Feature importance analysis derived from the Random Forest model, illustrated in Fig. 6, reveals a clear hierarchy of predictive influences among the input variables. PM₁₀ emerges as the most influential predictor, with an importance score of 12.7829, followed by NO₂ (5.8839) and PM₂.₅ (1.9800)^32^. This pronounced dominance of particulate matter variables over meteorological factors highlights the ability of the Random Forest model to identify the primary emission driven contributors to pollution regime formation in Cairo’s urban environment. The substantial importance of PM₁₀ underscores the critical role of coarse particulate matter and episodic dust storm events in shaping air pollution regimes, while the strong contribution of NO₂ reflects the persistent influence of traffic related emissions^35^. In contrast, meteorological variables exhibit comparatively lower importance scores, with near surface air temperature (1.1153) and wind speed (0.7962) ranking sixth and ninth, respectively. This pattern suggests that emission sources exert a stronger influence than atmospheric dispersion conditions in determining daily pollution regimes within this densely populated urban setting^46,47^. Figure 5 presents the relative importance of all environmental variables, clearly demonstrating the predominance of pollutant concentrations over meteorological factors in the Random Forest regime classification model^48^.

### Process level interpretation of NO₂ and PM₁₀ influence

Although both models achieved high accuracy, they differ in feature ranking: the Decision Tree highlights NO₂, whereas the Random Forest assigns greater importance to PM₁₀. To clarify this shift, we examined the joint influence of NO₂ and PM₁₀ using a pairwise partial dependence analysis for the dust regime (Cluster 4).

**Fig. 7:**
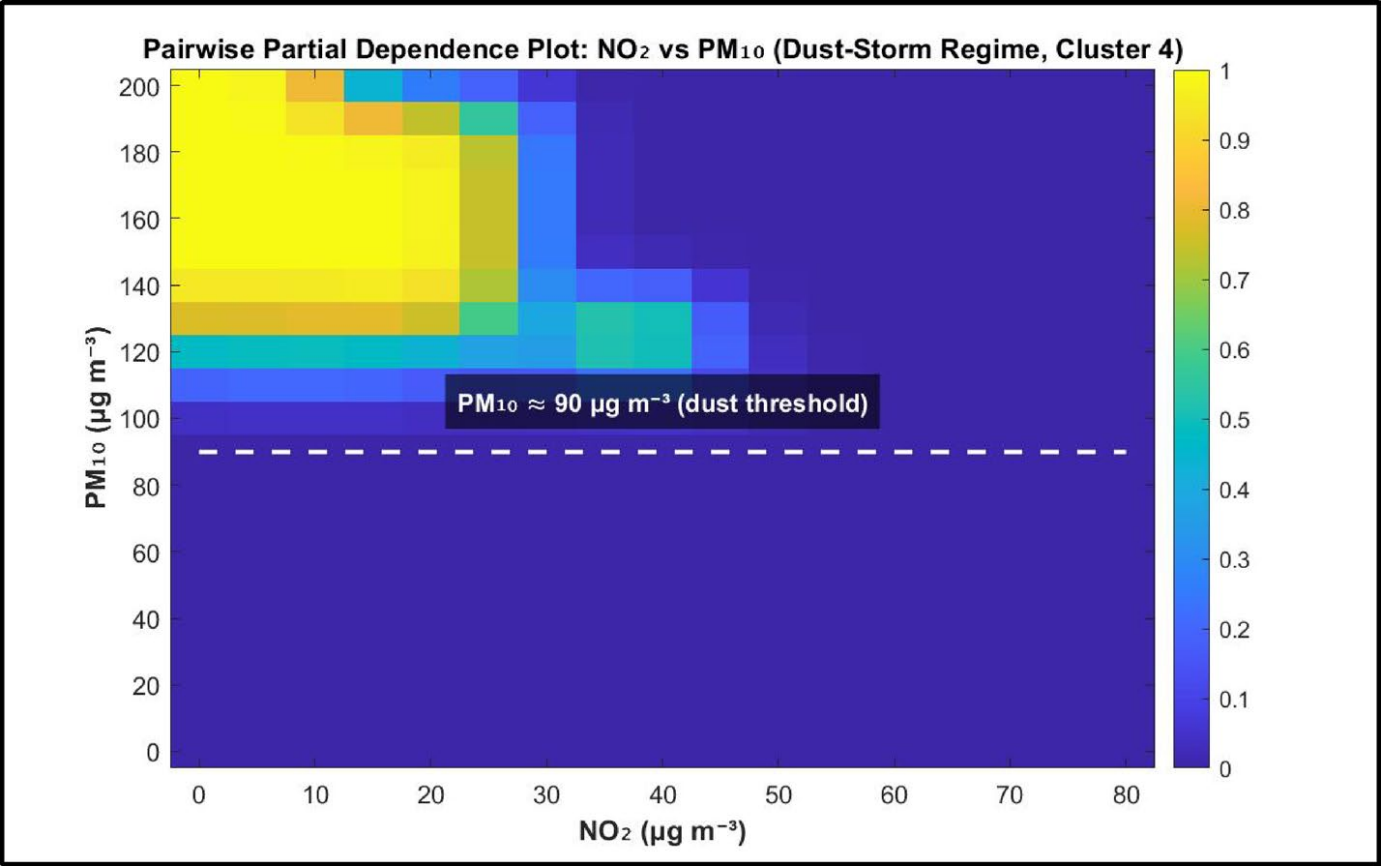
Partial dependence of dust-storm probability on NO₂ and PM₁₀.

The results Fig. 7 show a clear nonlinear threshold in PM₁₀. When PM₁₀ exceeds approximately 90 µg m⁻³, the probability of the dust regime increases sharply and becomes weakly sensitive to NO₂. Below this threshold, dust probability remains low. This indicates that PM₁₀ acts as a regime defining variable during dust events.

The Decision Tree prioritizes NO₂ due to its efficiency in separating traffic-related conditions, while the Random Forest captures the nonlinear threshold behavior of PM₁₀ through ensemble interaction modeling. Thus, the feature importance shift reflects genuine regime specific nonlinear dynamics rather than collinearity or model instability.

### Discussion

The integrated application of K-means clustering, Decision Tree classification, and Random Forest modelling provides a comprehensive interpretation of air pollution dynamics across Greater Cairo. Each method contributes a distinct analytical perspective: K-means identifies underlying air pollution regimes, the Decision Tree generates interpretable rule based structures linking environmental variables to regime membership, and the Random Forest model delivers enhanced predictive performance and classification stability.

The optimized K-means analysis reveals four clearly separated pollution regimes: very low pollution conditions (41.7%), low pollution conditions (33.4%), traffic dominated pollution (18.9%), and dust storm conditions (6.0%). Although the majority of the analyzed period (75.1%) is characterized by low to very low pollution regimes, persistent traffic emissions continue to account for a substantial fraction of pollution events, as reflected by elevated NO₂ concentrations. The dust storm regime, while representing a smaller proportion of observations, corresponds to environmentally critical episodes driven by episodic Saharan dust intrusions that result in sharp increases in PM₁₀ levels.

The Decision Tree classifier further supports the physical relevance of the identified regimes, achieving an accuracy of 93.10% following depth optimization. The dominance of NO₂ as the most influential predictor, followed by near surface air temperature and wind speed, highlights the combined role of traffic intensity and atmospheric stability in shaping day to day variability in pollution regimes.

Random Forest modelling provides a notable improvement in classification capability, reaching a peak accuracy of 97.49%, with a practical optimum of 97.43% achieved using an ensemble of 300 trees. Feature importance analysis identifies PM₁₀, NO₂, and PM₂.₅ as the dominant contributors, reinforcing the combined influence of dust intrusions, vehicular emissions, and fine particulate pollution on Cairo’s air pollution regimes. Compared with the Decision Tree baseline, the Random Forest model demonstrates reduced sensitivity to overfitting and offers more stable and operationally reliable regime classification.

The quantification of actual pollutant concentrations provides critical context for environmental management. The extreme PM₁₀ levels during dust storms (79.1–201.5 µg/m³) represent acute respiratory hazards requiring targeted public health advisories. Concurrently, persistent traffic related NO₂ elevation (11.5–30.8 ppb) underscores the need for sustained vehicular emission controls. These measured ranges transform the statistical clusters into actionable thresholds for early warning systems, directly supporting Cairo’s air quality management objectives.

Overall, the proposed hybrid analytical framework effectively distinguishes air pollution regimes, identifies their dominant drivers, and delivers a robust regime classification model suitable for regime based early warning and decision support applications. The strong agreement between unsupervised clustering and supervised classification confirms the physical consistency of the identified regimes and underscores the value of machine learning approaches for evidence driven urban air quality management in complex megacities such as Greater Cairo.

## Conclusion and future work

### Conclusion

This study applied an interpretable machine learning framework for the analysis of air pollution dynamics in Greater Cairo. The results indicate that combining unsupervised clustering with supervised classification provides a useful and complementary approach for understanding pollution regime behavior in a complex urban environment.

The main findings of the study can be summarized as follows:

- Four distinct air pollution regimes were identified in Greater Cairo, with low and very low pollution regimes accounting for approximately 75.1% of the analyzed period, while traffic dominated pollution (18.9%) and dust storm events (6.0%) represented important episodic conditions.
- The Random Forest classifier showed higher regime-classification performance, reaching a maximum accuracy of 97.49%, compared with 93.10% for the optimized Decision Tree model.
- Feature importance analysis revealed PM₁₀ as the dominant predictor in the Random Forest model, while NO₂ emerged as the most influential variable in the Decision Tree, highlighting the complementary strengths of the two algorithms in capturing dust related and traffic related pollution drivers.
- An ensemble size of 300 trees was identified as the optimal Random Forest configuration, providing an effective balance between computational efficiency and predictive accuracy (97.43%).

The consistent identification of traffic related signals (via NO₂) and dust related signals (via PM₁₀) across both clustering and classification analyses suggests that these are major drivers of air pollution regime variability in Greater Cairo. Overall, the framework provided an interpretable basis for pollution regime discovery and regime classification and may support air quality interpretation and future regime based monitoring efforts in complex urban settings.

These findings also indicate several directions for extending the present framework and improving future air pollution analysis and prediction systems.

From an operational perspective, the proposed regime based framework shows potential for supporting rapid air quality interpretation. Model inference itself requires only 0.01 s (as measured on our test dataset), demonstrating negligible computational latency.

Given the 3-hourly temporal resolution of CAMS reanalysis products and associated data processing pipelines, the effective system lead time operates on hourly timescales, primarily constrained by data refresh cycles rather than computational requirements.

The framework complements rather than replaces existing systems such as Cairo’s EMA-CAPPM (which provides 48-hour concentration forecasts) by delivering short-horizon, interpretable regime classifications particularly valuable during rapidly evolving pollution episodes.

### Limitations of the study

One limitation of this study is the reliance on CAMS reanalysis data rather than continuous ground based air quality measurements. Although CAMS provides spatially and temporally consistent atmospheric composition fields, global validation studies have reported regional biases over arid and dust prone regions, particularly related to aerosol speciation and coarse mode dust representation, which may affect absolute particulate matter concentration estimates.

In this work, CAMS data are used as a consistent representation of regional atmospheric variability rather than as point wise observations. The proposed framework focuses on identifying recurring air pollution regimes and relative temporal patterns, an approach that is less sensitive to systematic magnitude biases. Despite this limitation, the regime based analysis provides an interpretable basis for short term situational awareness and air quality assessment, particularly when considered alongside existing forecasting systems.

### Future research directions

Future work may extend the present research along several directions. First, incorporating longer multiyear datasets would enable improved characterization of long term climatic trends and seasonal variability in pollution regimes. Second, integrating satellite observations with ground based monitoring networks could enhance spatial representation and improve the robustness of regime identification in heterogeneous urban environments.

In addition, exploring deep learning architectures may offer improved capability for capturing complex temporal dependencies and supporting high resolution forecasting of pollution regimes. The development of real time, operational air pollution monitoring and prediction platforms based on the proposed framework also represents a promising avenue for practical implementation. Finally, applying the framework to other rapidly urbanizing regions facing similar environmental challenges would help assess its generalizability and broader applicability. Collectively, these extensions may help further evaluate the usefulness of machine learning based approaches for urban air quality analysis and environmental decision support.

## Data Availability

Data for this study are publicly available from the Zenodo repository at https://doi.org/10.5281/zenodo.19544415.
